# Generation of a Soluble African Horse Sickness Virus VP7 Protein Capable of Forming Core-like Particles

**DOI:** 10.3390/v14081624

**Published:** 2022-07-26

**Authors:** Shani Bekker, Henk Huismans, Vida van Staden

**Affiliations:** Department of Biochemistry, Genetics and Microbiology, University of Pretoria, Hatfield 0083, South Africa; shani.bekker@gmail.com (S.B.); hhuismans@icon.co.za (H.H.)

**Keywords:** African horse sickness virus, VP7, mutagenesis, solubility, crystalline particle, core-like particle, virus-like particle, vaccine, equine

## Abstract

A unique characteristic of the African horse sickness virus (AHSV) major core protein VP7 is that it is highly insoluble, and spontaneously forms crystalline particles in AHSV-infected cells and when expressed in vitro. The aggregation of AHSV VP7 into these crystals presents many problems in AHSV vaccine development, and it is unclear whether VP7 aggregation affects AHSV assembly or contributes to AHSV pathogenesis. Here, we set out to abolish VP7 self-assembly by targeting candidate amino acid regions on the surface of the VP7 trimer via site-directed mutagenesis. It was found that the substitution of seven amino acids resulted in the complete disruption of AHSV VP7 self-assembly, which abolished the formation of VP7 crystalline particles and converted VP7 to a fully soluble protein still capable of interacting with VP3 to form core-like particles. This work provides further insight into the formation of AHSV VP7 crystalline particles and the successful development of AHSV vaccines. It also paves the way for future research by drawing comparisons with similar viral phenomena observed in human virology.

## 1. Introduction

African horse sickness (AHS) is a devastating disease of equids with up to 95% mortality in susceptible horses. AHS is caused by an orbivirus of the family *Reoviridae* known as African horse sickness virus (AHSV). Along with bluetongue virus (BTV), AHSV is one of the most economically important members of the *Orbivirus* genus. Orbiviruses are complex, non-enveloped viruses, with a characteristic dsRNA genome contained within an icosahedral core particle made up of the core proteins VP7 and VP3. The core particle is encapsidated by the structural proteins VP2 and VP5, which make up the outer capsid layer of the virion [1,2,3].

The clear overall similarities in the structure and replication strategies of viruses within the *Orbivirus* genus have allowed for BTV to be the prototype of all orbiviruses. AHSV, however, differs significantly from BTV in that the major core protein of AHSV, VP7, has the unique characteristic of assembling into distinctive insoluble, flat, hexagonal crystalline particles during AHSV infection [4,5]. Despite having a sequence similarity of 70% with AHSV VP7 [6], BTV VP7 is highly soluble and does not assemble into any morphological entities when expressed in the absence of other virus proteins [7,8]. It has been suggested that the formation of AHSV VP7 crystalline particles likely sequesters available VP7 into these particles, thus preventing their incorporation into newly forming cores [9]. This is postulated to have a negative impact on the efficiency of AHSV core assembly and the resulting replication rate. It is not clear how VP7 aggregation affects AHSV particle assembly or whether the presence of these crystals could contribute to the cellular pathogenesis caused by AHSV infection, which is more severe than that of BTV. Virus protein aggregation has been associated with a range of human diseases such as COVID-19 [10,11,12,13], hepatitis B [14], and influenza [15]. It is, therefore, necessary to identify which forces drive the formation of these crystalline particles to better understand the evolution, pathogenesis, and replication of AHSV.

Apart from our desire to understand the role of VP7 crystalline particle formation, the negative effects of AHSV VP7 aggregation are evident in AHSV vaccine development. The need for alternative next-generation BTV and AHSV vaccines has sparked new vaccine developments such as recombinant subunit vaccines, and the generation of recombinant vaccine viruses via reverse genetics [16,17,18,19]. The most successful BTV subunit vaccine is based on the co-expression of the four structural proteins of BTV, i.e., VP2, VP5, VP7, and VP3, which results in the assembly of empty virus-like particles (VLPs) [20]. VLPs are strong vaccine candidates as they mimic virions but lack the viral genome and, therefore, the ability to replicate. Their safety and efficacy have made VLPs attractive vaccine candidates for many viral diseases [21,22,23]. The vaccination of sheep with multiple serotypes of BTV VLPs has shown long-lasting protection against homologous BTV challenge with evidence of partial heterologous virus cross-protection [24,25]. Furthermore, BTV VLPs expressed in plants have been shown to be as effective as a live attenuated virus in protecting sheep from infection [26]. 

VLPs are also currently used as therapeutic proteins and as safe and effective vaccines for a variety of human diseases such as hepatitis B [27] and cervical cancer [28]. As a cervical cancer vaccine, human papillomavirus (HPV) VLPs offer high immunoprotection against the targeted strains of HPV [28,29]. Viral capsid protein aggregation, which results in polymorphic or amorphous aggregate structures, has been reported to often be in competition with capsid assembly for the production of several types of VLP such as HPV [30,31,32,33]. Similarly, the development of a successful AHSV VLP vaccine has not been possible due to inefficient AHSV VLP yields [34]. The diminished yields of AHSV VLPs are attributed to the limited availability of AHSV VP7 during core-like particle (CLP) assembly due to the preferential aggregation of AHSV VP7 into crystalline particles [34,35]. Moreover, VP7 is highly conserved within its serogroup and is the most abundant major immunogenic serogroup-reactive protein of most orbiviruses [36]. VP7 has also been shown to contain both B- and T-cell epitopes across multiple orbiviruses [37,38,39,40,41]. For these reasons, VP7 from both BTV and AHSV has been tested as a subunit vaccine candidate and has offered protection against virulent virus challenge in immunised animals in numerous studies [42,43,44,45]. Like a potential AHSV CLP or VLP vaccine, however, VP7 expression or delivery to the host cell is complicated due to the aggregation of VP7 proteins into these particulate structures. The aggregation of AHSV VP7 also previously posed problems when AHSV VP7 was tested for its use as an antigen display system via the insertion of foreign epitopes into the VP7 top domain [46]. Given the evident negative effects of AHSV VP7 crystalline particle formation in AHSV vaccine development, a soluble AHSV VP7 will provide a vast number of possibilities for the development of improved AHSV vaccines. 

Here, we aimed to abolish AHSV self-assembly into crystalline particles by targeting trimer–trimer interactions and converting AHSV VP7 into a fully soluble protein. We previously suggested that the unique ability of AHSV VP7 to aggregate into particles is the result of AHSV VP7 self-assembly, caused by trimer–trimer interactions, driven by residues on the surface of the AHSV VP7 trimer that functionally differ from the corresponding BTV VP7 residues [47]. In this study, we selected six regions of such residues as candidates that may drive AHSV VP7 self-assembly and designated these regions “45”, “131”, “136”, “172”, “193”, and “276”. These regions were targeted for substitution to the BTV VP7 corresponding region via site-directed mutagenesis and six modified VP7 proteins were generated. The role of each region in AHSV VP7 self-assembly was tested by analysing VP7 mutant trimer formation and solubility. It was found that the combined amino acid substitutions in the “276” region, i.e., Pro276His, Arg328Ala, Val333Asn, Ala334Pro, Pro335Met, Val336Pro, and Gln338Pro, converted AHSV VP7 into a fully soluble protein. It was also found that this modified VP7 successfully interacts with VP3 to form CLPs. The results obtained here allow us to understand the mechanism of AHSV VP7 crystalline particle formation, and the potential impact of this particle formation on AHSV core assembly. This study has also resulted in the generation of a patented soluble version of AHSV VP7, which can be used in future AHSV diagnostics and vaccine developments such as improved AHSV VLPs.

## 2. Materials and Methods

### 2.1. Cells and Viruses

*Spodoptera frugiperda* (Sf9) cells were maintained at 28 °C, suspended in TC-100 insect medium (Lonza, Basel, Switzerland) with nonessential amino acids supplemented with 10% (*v*/*v*) foetal calf serum and penicillin/streptomycin (Lonza, Basel, Switzerland), and fungizone (Sigma-Aldrich^®^, Merck, Darmstadt, Germany). Recombinant baculoviruses were generated using the Bac-to-Bac^®^ Baculovirus expression system (Thermo Fisher Scientific, Waltham, MA, USA) and were propagated in Sf9 insect cells according to the manufacturer’s instructions.

### 2.2. Generation of Modified VP7 Proteins

Six mutant VP7 proteins were generated and expressed by means of the Bac-to-Bac^®^ Baculovirus expression system (Thermo Fisher Scientific, Waltham, MA, USA). A wild-type (WT) AHSV-9 VP7 gene contained within the baculovirus donor vector, pFastBac™ (pFB-VP7) [35], was used as a template for the construction of the six modified VP7 genes. To introduce site-specific nucleotide changes across vast regions of the gene, dsDNA fragments containing the desired nucleotide mutations (for each construct) in the VP7 sequence, spanning from the first nucleotide change to the last nucleotide change, were designed (gBlock Gene Fragments, Integrated DNA Technologies, Coralville, IA, USA) and inserted into the pFB-VP7 backbone using the In-Fusion^®^ HD Cloning kit (Clontech^®^, Takara Bio Inc., Shiga, Japan). Briefly, the dsDNA fragments were designed to include 15 bp homologous ends to the VP7 sequence (Table 1); meanwhile, each respective pFB-VP7 vector backbone was linearised via inverse PCR using primers, which contained the same 15 bp homologous ends in their 5′ ends as the DNA fragments to be inserted. Each dsDNA fragment was then fused with its respective linearised pFB-VP7 via the In-Fusion^®^ enzyme according to the manufacturer’s instructions.

Once constructed, the sequence of each modified VP7 gene was confirmed and used to generate and amplify recombinant baculoviruses according to the manufacturer’s instructions. Briefly, purified DNA from pFastBac™ donor plasmids containing the gene of interest were transformed into DH10Bac™ *E. coli* cells; this was carried out for transposition of the gene of interest under the control of *Autographa californica* multiple nuclear polyhedrosis virus (AcMNPV) polyhedrin promoter (flanked by Tn7 transposons) into the baculovirus shuttle vector (bacmid) DNA. Purified recombinant bacmid DNA containing each gene of interest was then transfected into Sf9 cells to produce recombinant baculovirus. A recombinant baculovirus containing AHSV-9 VP3 was similarly generated using a previously available pFB-VP3 construct [35].

### 2.3. Protein Expression, SDS-PAGE, and Western Blot Analysis

For the expression of each modified VP7 gene, Sf9 monolayers were infected with recombinant baculovirus at an MOI of 5 to 10 and incubated at 28 °C for 48 h. Cells were then harvested via centrifugation at 800× *g* for 5 min. Cell pellets were washed in 1× phosphate-buffered saline (PBS) (137 mM NaCl, 2.7 mM KCl, 4.3 mM Na_2_HPO_4_.2H_2_O, 1.4 mM KH_2_PO_4_, pH 7.3) and lysed via 30 min incubation at 4 °C in lysis buffer (10 mM Tris-HCl pH 8.0, 50 mM EDTA, 10 mM NaCl, 0.5% [*v*/*v*] Nonidet^®^ P-40); then homogenisation was performed by passing the cell pellets through a 22G needle twenty times. Cell lysates were analysed via SDS-PAGE or Western blot as described previously [9]. Briefly, samples were incubated in 3× protein solvent buffer (10% [*v*/*v*] β-mercaptoethanol, 4% [*w*/*v*] SDS, 20% [*v*/*v*] glycerol, 125 mM Tris-HCl pH 6.8, and 0.002% [*w*/*v*] bromophenol blue) at 95 °C or at room temperature (for identification of VP7 trimers) for 5 min and resolved using either 8 or 10% SDS-PAGE; this was followed by staining with Coomassie brilliant blue. For Western blot analysis, proteins were transferred from SDS-PAGE gels to a Hybond™ C extra nitrocellulose membrane (Amersham Biosciences, Amersham, UK) via standard blotting procedures. The membrane was incubated in customised rabbit polyclonal antibody at a concentration of 1:100 in 1% blocking solution (1% [*w/v*] milk powder in PBS). A primary antibody against AHSV VP3 was produced in rabbits by GenScript Biotech Corp (Piscataway, NJ, USA) (complete affinity-purified peptide polyclonal antibody) and diluted 1:100 in 1% blocking solution. A custom polyclonal antibody for AHSV VP7 used in this study was kindly supplied by Prof. J. Theron (University of Pretoria). These antibodies were used at a concentration of 1:100 in 1% blocking solution. Peroxidase-conjugated Protein A, diluted 1:10,000, was used for detection.

### 2.4. Solubility Assay

Modified VP7 proteins were expressed and harvested as described in Section 2.3. Cell lysates were centrifuged at 16,000× *g* for 30 min. The resulting pellets (resuspended in 400 μL 0.01 M STE buffer) and supernatants were analysed via SDS-PAGE and Western blotting. Further solubility analysis was performed by analysing VP7 fractions from sucrose gradient ultracentrifugation. Sf9 cell monolayers (2 × 10^7^ cells/flask) were infected with VP7 recombinant baculovirus (at an MOI of 5). Cells were incubated at 28 °C for 48 h, and harvested as described in Section 2.3. Nuclei and debris were removed via centrifugation at 100× *g* for 5 min before being loaded onto a sucrose discontinuous step-gradient (1 mL 40% sucrose layered onto 1 mL 66% sucrose in 0.2 M Tris-HCl with 140 mM NaCl) and ultracentrifuged at 85,000× *g* for 3 h at 4 °C. Six fractions (500 μL) were collected, and the pellet was resuspended in 100 μL 0.2 M Tris-HCl. A sample from each fraction (20 μL) and the pellet (10 μL) was then analysed via Western blot analysis.

### 2.5. Immunofluorescence Microscopy

Sf9 cell monolayers, grown on coverslips in 24-well plates, were infected with recombinant baculovirus at an MOI of 10 and incubated at 28 °C for 48 h. Cells were washed thrice in 1 × PBS and fixed in a 1:1 methanol:acetone solution at 4 °C for 5 min. Cells were washed again and incubated in 5% blocking solution for 30 min at room temperature, followed by incubation with AHSV VP7 (guinea pig) (available at the start of this investigation [46]); this was pre-adsorbed against wild-type baculovirus-infected Sf9 cells or VP3 (rabbit) primary antibody (Section 2.3) for 1 to 16 h. Cells were washed three times with wash buffer (0.5% (*v*/*v*) Tween^®^ 20 in PBS), then, once with PBS, and incubated for 1 h using Alexa Fluor 488 anti-guinea pig- or Alexa Fluor 633 anti-rabbit conjugated secondary antibody (Thermo Fisher Scientific, Waltham, MA, USA) at a concentration of 1:250. Cells were washed again and stained with 10 μg/mL DAPI (4′,6-Diamidine-2′-phenylindoledihydrochloride) in 1% blocking solution for 10 min. Cells were washed once with PBS before being mounted onto glass slides using Vectashield Mounting Medium (Vector Laboratories, Newark, CA, USA). Slides were analysed via laser scanning confocal microscopy (LSCM) using a Zeiss LSM 510 Meta confocal microscope. Colocalisation analysis was conducted using the Zeiss ZEN 3.3 Blue edition (Carl Zeiss Microscopy, Oberkochen, Germany) software package. To determine fluorescence intensity profiles, a line was drawn across the region of interest and a fluorescence intensity plot was obtained using the ZEN software.

### 2.6. Production and Purification of Recombinant Core-like Particles

Sf9 cell monolayers (2 × 10^7^ cells/flask) were co-infected with VP7 recombinant baculovirus (at an MOI of 5) and VP3 recombinant baculovirus (at an MOI of 10). Cells were incubated, harvested, clarified, loaded onto a sucrose discontinuous step-gradient, and underwent ultracentrifugation as described in Section 2.4. CLPs were purified by pooling fractions 2 and 3 of the discontinuous gradient and diluting them to 2 mL using 0.2 M Tris-HCl. These fractions were layered over a 1 mL 40% sucrose cushion followed by ultracentrifugation at 100,000× *g* for 2 h at 4 °C. Pellets containing purified CLPs were resuspended in 100 μL 0.2 M Tris-HCl. The purified CLPs were adsorbed onto copper Formvar carbon-coated 400-mesh grids by floating the grids on droplets of the sample for 3 min. After being dried, the particles were negatively stained for 3 min on droplets of 2% uranyl acetate. Grids were examined in a JOEL JEM-2100F field-emission transmission electron microscope (TEM).

### 2.7. Protein Three-Dimensional Structure Modelling 

A homology model of the AHSV VP7 trimer was used to create three-dimensional (3D) models using the VMD (Visual Molecular Dynamics) software package [48] and its extension, MultiSeq [49], as described previously [47]. VMD was developed by the Theoretical and Computational Biophysics Group at the Beckman Institute for Advanced Science and Technology at the University of Illinois Urbana-Champaign (http://www.ks.uiuc.edu/Research/vmd/ (accessed on 1 March 2013)).

### 2.8. Protein Sequence Alignments

Multiple sequence alignments were calculated based on percent identity, and visualised using Jalview [50]. The Genbank accession numbers for each sequence are previously described [47].

## 3. Results

### 3.1. Selection of Regions on the VP7 Trimer Surface for Site-Directed Mutagenesis and Expression of Modified VP7 Proteins

**Figure 1:**
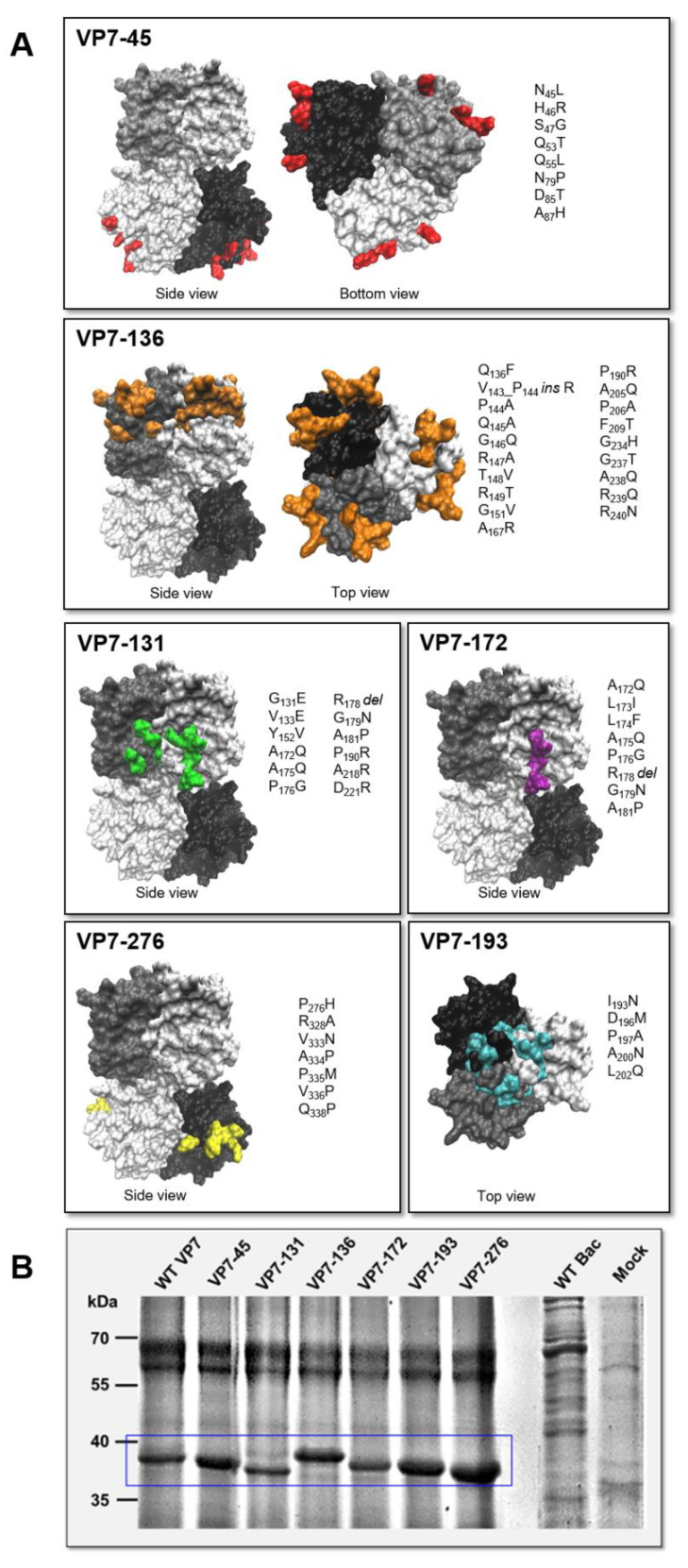
Generation and expression of modified AHSV VP7 proteins: (**A**) Summary of VP7 modifications for construction of the VP7 mutant proteins. Three-dimensional models of the AHSV trimer are depicted with viewpoint indicated, i.e., side, top, or bottom view. Each monomer of the trimer is coloured grey, white, or black. The amino acid substitutions are highlighted in colour in each trimer, and the substitutions are illustrated on the **right** of each trimer model. (**B**) Confirmation of the expression of modified AHSV VP7 proteins using 12% SDS-PAGE. Insect cells were infected at an MOI of 10 with recombinant baculoviruses for each construct and harvested 48 hpi. Proteins banded at the expected size of 38 kDa were comparable to WT VP7 (box). Wild-type baculovirus (WT Bac) and mock-infected cell preparations were used as controls.

To determine whether the surface properties of the AHSV VP7 trimer are responsible for trimer–trimer interactions that result in VP7 self-assembly into crystalline particles, we targeted previously identified candidate AHSV VP7 surface amino acids for mutation via site-directed mutagenesis. Recently, amino acids present on the surface of the AHSV VP7 trimer were mapped and designated as candidates for AHSV VP7 self-assembly, resulting from their physiological differences to the corresponding BTV VP7 amino acids [47]. By grouping these amino acids based on their proximity on the surface of the trimer, nine regions were identified as candidates that could potentially drive AHSV VP7 aggregation [47]. Here, we targeted these regions for substitution to the corresponding BTV VP7 regions and tested the resulting mutant proteins’ solubility and ability to form aggregates and/or self-assemble into crystalline particles. By combining our previous data [47] a total of six regions were carefully selected for site-directed mutagenesis to generate the mutants designated VP7-45, VP7-131, VP7-136, VP7-172, VP7-193, and VP7-276 (Figure 1A). In each mutant, between 5 and 19 amino acid substitutions corresponding to a specific region on the surface of the VP7 trimer were created, and the modified proteins were expressed using the baculovirus expression system (Figure 1B). Once recombinant protein expression was confirmed, the modified VP7 proteins were analysed for the effects of each modification.

### 3.2. Solubility Analysis of Modified AHSV VP7 Proteins

**Figure 2:**
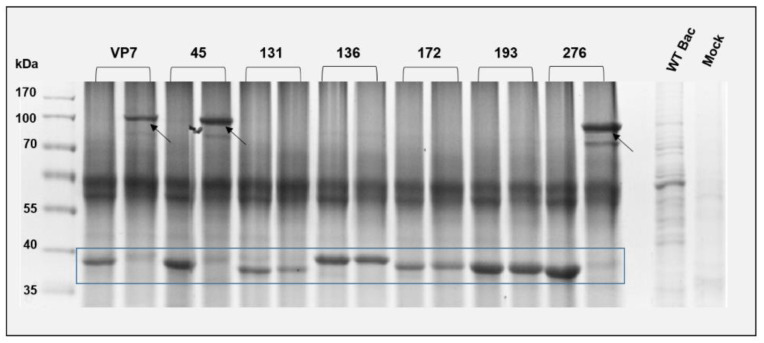
Trimerisation assay of modified VP7 proteins: A modified SDS-PAGE of heat-denatured (**left**) and non-denatured (**right**) samples from recombinant baculovirus-infected cell preparations of each mutant AHSV VP7 protein were run alongside one another. VP7 monomers are indicated by the ~38 kDa bands in the box. Multimers are indicated by high-molecular-weight bands (~100 kDa) in non-denatured samples comparable to WT VP7 (arrows). Wild-type baculovirus (WT Bac) and mock-infected cell preparations were used as controls.

The VP7 modifications introduced here included the substitution of multiple amino acids in each construct. Alterations such as these may have a negative impact on the folding of VP7. Results from Figure 1B indicated that all modified proteins were able to form 38 kDa monomeric bands comparable to WT VP7. To determine the effect of these modifications on VP7 folding and thus normal trimer formation, a crude trimerisation assay was performed as described previously [9]. Sf9 cells were infected with recombinant baculoviruses of each construct. Cells were harvested 48 h post infection (hpi), lysed, washed, and processed for SDS-PAGE with and without heat denaturation (Figure 2). Results showed that non-denatured VP7-45 and VP7-276 formed high molecular weight multimers comparable to WT VP7 (Figure 2 arrows), indicating the presence of stable trimer formation. No multimers (only monomers) were present in non-denatured samples of VP7-131, VP7-136, VP7-172 and VP7-193. All modified proteins formed 38 kDa monomeric bands in denatured samples (Figure 2 box). Interestingly, only those proteins with modifications in their bottom domain, i.e., VP7-45 and VP7-276, were able to form stable trimers comparable to WT VP7 whereas the rest with modifications in their top domains did not. The presence of multiple bands in each case is due to the non-denatured state of the proteins that likely aggregate to form larger molecular weight bands than expected and due to the instability of these proteins in the presence of SDS. This type of trimerization assay is only used for the positive identification of highly stable trimers comparable to WT VP7, the absence of a high molecular weight band is therefore not a true indication of the inability of VP7-131, VP7-136, VP7-172 and VP7-193 to form trimers; it could be possible that they form less stable trimers undetectable here. For the purposes of this study, it was not necessary to perform further trimerisation analysis.

**Figure 3:**
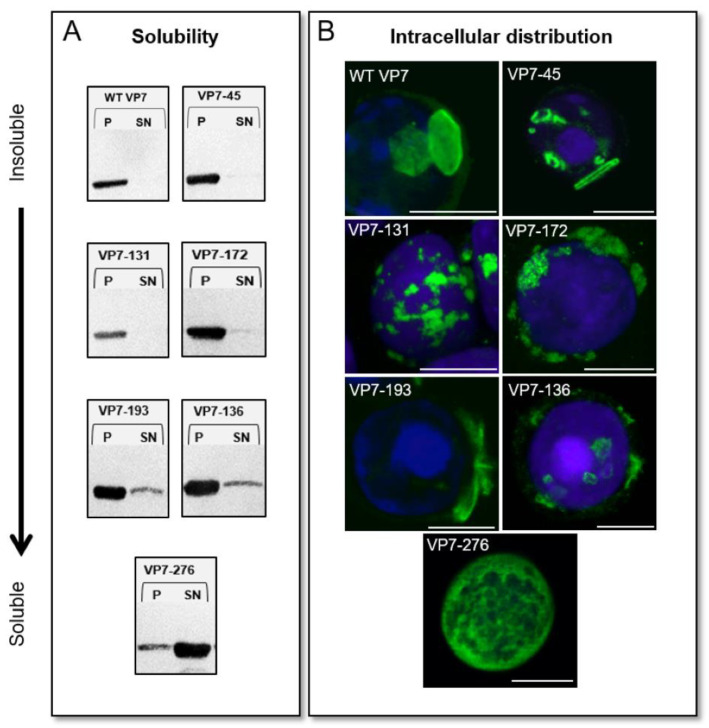
Analysis of the self-assembly and solubility of modified AHSV VP7 proteins: Proteins are arranged according to their solubility (**A**) and intracellular distribution (**B**), from WT VP7 being the most insoluble and crystalline, to VP7-276 being the most soluble and diffuse. (**A**) Solubility assay of modified VP7 proteins. Western blot analysis of pellet (P) and supernatant (SN) fractions of equal amounts of each VP7 mutant protein using anti-VP7 antibody. Recombinant baculovirus-infected cells were harvested 48 hpi, lysed, and washed, and soluble and insoluble fractions were separated via centrifugation. (**B**) Intracellular distribution of VP7 mutant proteins using indirect immunofluorescence microscopy. Recombinant baculovirus-infected insect cells expressing VP7 mutant proteins were fixed 48 hpi and processed for immunofluorescence microscopy. VP7 proteins were detected via anti-VP7 primary antibody and Alexa Fluor 488 conjugated secondary antibody (green). Nuclei were stained using DAPI (blue). Slides were analysed using LSCM. Scale bars represent 10 μm.

To further analyse the effect of these modifications on AHSV VP7 self-assembly, the solubility profiles and the intracellular distribution of each of the modified VP7 proteins were analysed and compared to WT VP7. AHSV VP7 is highly insoluble, and most of the protein self-assembles into insoluble aggregates known to be in the form of crystalline particles [9,51], which are easily purified by centrifugation [46,51]. If trimer–trimer interactions were responsible for AHSV VP7 self-assembly into these crystalline particles, then successful disruption of these interactions by the surface amino acid modifications made here would increase the solubility of AHSV VP7 as it would prevent aggregation. To analyse the solubility of these modified VP7 proteins, the soluble and insoluble fractions from each protein lysate were obtained. A basic solubility assay was performed whereby recombinant baculovirus-infected cell lysates were washed, lysed, and subjected to centrifugation at 16,000× *g* for 30 min to separate crude insoluble material in the pellet from soluble components in the supernatant. Each pellet (P) and supernatant (SN) was analysed via Western blot using the anti-VP7 antibody (Figure 3A). To examine VP7 intracellular distribution, Sf9 cells were seeded onto coverslips and infected with recombinant baculovirus expressing each modified VP7 separately, and processed at 48 hpi for laser scanning confocal microscopy (LSCM) visualisation. Three-dimensional reconstructions of each construct were created from Z-stack images (Figure 3B). Figure 3 shows the combined data from the solubility assay (Figure 3A derived from Figure S1) coupled with representative immunofluorescence microscopy (Figure 3B—see Figure S2 for more examples) for each modified VP7 protein arranged according to degree of solubility. 

When examining the intracellular distribution, WT VP7 aggregated into the characteristic flat, hexagonal-shaped crystalline particles (Figure 3B); these can be concluded to be insoluble based on the protein only being detected in the insoluble fraction for WT VP7 (Figure 3A). Like WT VP7, VP7-45 was insoluble and formed flat particles intracellularly. The modifications made in VP7-45, therefore, had no observed effect on VP7 self-assembly. Other similarly insoluble proteins included VP7-131 and VP7-172; however, they did not form characteristic crystalline particles, but rather, numerous punctate foci of irregular shape throughout the cell. The modifications of VP7-131 and VP7-172, therefore, slightly affected VP7 self-assembly into crystalline particles, but still caused VP7 to self-assemble into other aggregated insoluble structures. The solubility assays showed that VP7-136 and VP7-193 had some protein present in the soluble supernatant fraction, but the majority was in the insoluble pellet (Figure 3A). VP7-193 formed long, thin, rigid, spindle-like structures, while VP7-136 formed several crystalline-like foci throughout the cell, with the majority of the protein present in the insoluble pellet fraction (Figure 3A); this indicates that the forces that drive trimer–trimer interactions were still at play for these constructs. Thus, the modifications of VP7-45, VP7-131, VP7-136, VP7-172, and VP7-193 did not significantly affect VP7 self-assembly into particulate structures. 

The most significant result was that observed for VP7-276. Unlike any of the other modified proteins, the majority of VP7-276 migrated to the soluble fraction (Figure 3A). The complementary intracellular distribution of VP7-276 was evenly distributed throughout the cell, with no formation of any discrete morphological structures (Figure 3B). From these results, it was concluded that the modifications of VP7-276 successfully disrupted the AHSV VP7 trimer–trimer interactions that lead to VP7 aggregation and converted AHSV VP7 into a soluble protein. These results were confirmed by analysing the solubility profiles of each protein on a sucrose gradient (data not shown).

### 3.3. Function Analysis of the Soluble AHSV VP7-276 Protein

**Figure 4:**
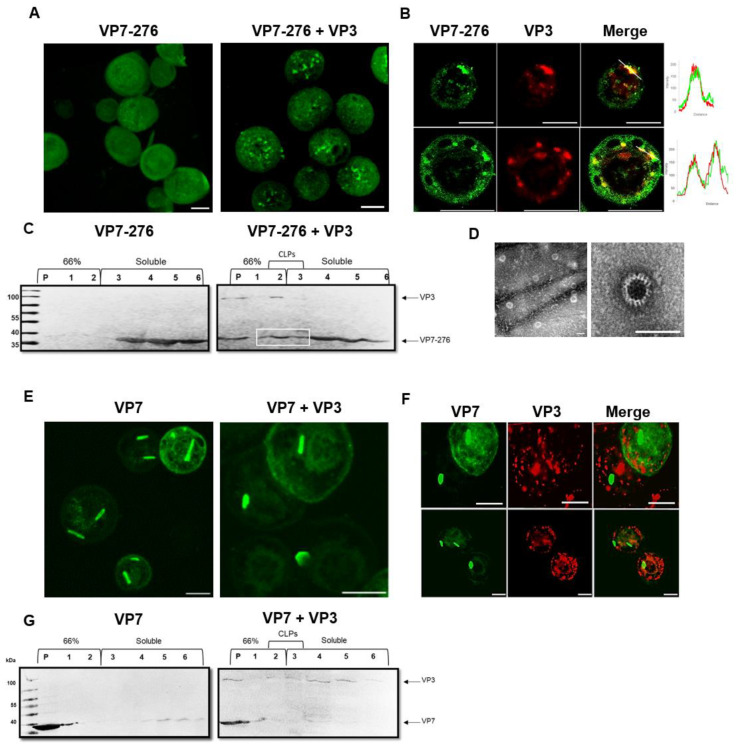
Examination of the VP7-276 interaction with VP3 to form CLPs: (**A**) Intracellular distribution of VP7 in cells expressing VP7-276 alone (**left**) and in the presence of VP3 (**right**). Cells were fixed at 48 hpi and labelled with anti-VP7 primary antibodies and Alexa Fluor 488 conjugated secondary antibodies. (**B**) Colocalisation analysis of the VP7-276 interaction with VP3 by indirect immunofluorescence and LSCM. Cells co-expressing VP3 and VP7-276 were co-labelled with primary antibodies against VP3 (rabbit) and VP7 (guinea pig), and secondary antibodies Alexa Fluor 633 conjugated anti-rabbit (red) and Alexa Fluor 488 conjugated anti-guinea pig (green), respectively. Two examples given, each with split images. Colocalisation of VP7-276 and VP3 in merged images is observed in yellow and was confirmed by measuring the fluorescence intensity of each channel along the white line drawn across a region of interest. Plots of fluorescence intensity vs. distance of the green and red channels are represented on the **right**. Scale bars represent 10 μm. (**C**) Western blot analysis of the solubility profile of VP7-276 (**left**) and VP7-276 in the presence of VP3 (**right**) from a 40% and 66% sucrose discontinuous step-gradient. Pellets (P) and 500 μL fractions (1 to 6), collected from a 40% and 66% sucrose discontinuous step-gradient, are indicated on the blots. VP7 (38 kDa) and VP3 (103 kDa) proteins were detected by primary antibodies as described in Section 2.3. The pellet (P) and fraction 1 represent insoluble fractions (66% sucrose), fractions 2 to 3 represent banding of CLPs (white box) at the interface, and 4 to 6 represent soluble fractions made up of 40% sucrose and cell lysate. (**D**) TEM micrographs of CLP(s) (~70 nm) formed by VP7-276 and VP3 co-expression. CLPs were isolated from fractions 2 and 3 of 40% and 66% sucrose discontinuous step-gradients, which had been purified through a 40% sucrose cushion. Purified CLPs were absorbed onto carbon-coated copper 400-mesh EM grids for 5 min, stained with 1% uranyl acetate, and visualised using a JEOL JEM-2100F field-emission transmission electron microscope. **Left** micrograph indicates a larger field, while **right** micrograph indicates an individual CLP for clarity. Scale bars represent 100 nm. (**E**–**G**) Experiments from A to C were repeated for WT VP7 in the presence and absence of VP3.

Being the major core protein of AHSV, the role of VP7 is to assemble on the scaffold provided by the minor core protein VP3 to form the AHSV core during virus assembly. To determine whether the modifications made in the VP7-276 protein affect VP7 functionality, the ability of the soluble VP7-276 mutant protein to interact with VP3 to form core-like particles was examined. First, we investigated whether VP7-276 interacts with VP3, by analysing the effect of the presence of VP3 on the homogenous intracellular distribution of VP7-276. Insect cells were infected with recombinant baculovirus to express either VP7-276 alone, or co-infected to express VP3 and VP7-276. These cells were incubated for 48 h, fixed, labelled with anti-VP7, and examined by means of LSCM. It was found that VP7-276 drastically changed from its diffuse distribution in the absence of VP3 to form concentrated cytoplasmic foci in the presence of VP3 (Figure 4A). To confirm whether VP7-276 was interacting with VP3, these proteins were co-labelled with anti-VP7 and anti-VP3 antibodies with the appropriate secondary antibodies, and their colocalisation was analysed. The results showed that the foci formed by VP7-276 in the presence of VP3 colocalised with VP3, confirming VP7-276 interaction with VP3 at these sites (Figure 4B). Colocalisation was confirmed, as shown in Figure 4B, where a white line is drawn across a region of interest in the merged image; the fluorescence intensity data for each channel across this line were obtained via Zeiss ZEN software. The fluorescence intensity data are displayed as plots alongside the merged images (Figure 4B right). In this instance, non-random colocalisation is indicated by the red and green fluorescence profiles that peaked at the same place.

To determine whether this interaction results in the formation of CLPs, we set out to synthesise and isolate CLPs using the soluble VP7-276. CLPs have previously been purified via banding at the interface of a 40% and 66% sucrose discontinuous step-gradient [52]. To synthesise CLPs, Sf9 cells were co-infected with recombinant baculoviruses encoding VP7-276 and VP3. After cell debris and nuclei were removed, samples were loaded onto a discontinuous 40% and 66% sucrose step-gradient and subjected to ultracentrifugation. Six fractions (including the pellet) were collected, and the solubility profiles of VP7-276 and VP3 were analysed via Western blot using anti-VP7 and anti-VP3 antibodies (Figure 4C). The distribution of VP7-276 was observed to shift from soluble fractions 4–6 when expressed alone, toward fractions 2–3 in the presence of VP3. Fractions 2–3 represent the gradient interface where CLPs are reported to band, which is confirmed by the migration of the majority of VP3 to fraction 2 (Figure 4C). These results correspond to the shift in intracellular distribution observed in Figure 4A, and further indicate that VP7-276 actively interacts with VP3.

To confirm that the VP7-276-VP3 interaction results in the formation of proper AHSV CLPs, CLPs were isolated and purified from fractions 2 to 3 of the gradient (Figure 4C). The fractions were pooled; then, the CLPs were collected via high-speed centrifugation and prepared for transmission electron microscope (TEM) visualisation. Isolated 70 nm CLPs were observed in the CLP sample preparations of VP7-276 co-expressed with VP3 (Figure 4D). These CLPs were morphologically identical to those previously reported for WT VP7 co-expressed with VP3 [34]. Taken together, these results indicate that the soluble VP7-276 mutant successfully interacts with VP3 to assemble into proper AHSV CLPs.

As a reference, the intracellular distribution and solubility profiles of WT VP7 and VP3 were examined in a similar manner to that described above (Figure 4E–G). Wild-type VP7 distribution did not notably change in the presence of VP3 (Figure 4E) compared to VP7-276 (Figure 4A), and the majority of VP7 was aggregated into crystalline particles with no notable colocalisation observed with VP3 (Figure 4F). The lack of association of WT VP7 with VP3 was confirmed via 3D reconstruction using Z-stack analysis (Video S1A,B). Furthermore, the majority of VP7 remained in insoluble fractions, with little notable change in the presence of VP3 (Figure 4G) compared to VP7-276 (Figure 4C).

### 3.4. Analysis of the Amino Acids Responsible for AHSV VP7 Self-Assembly into Crystalline Particles

**Figure 5:**
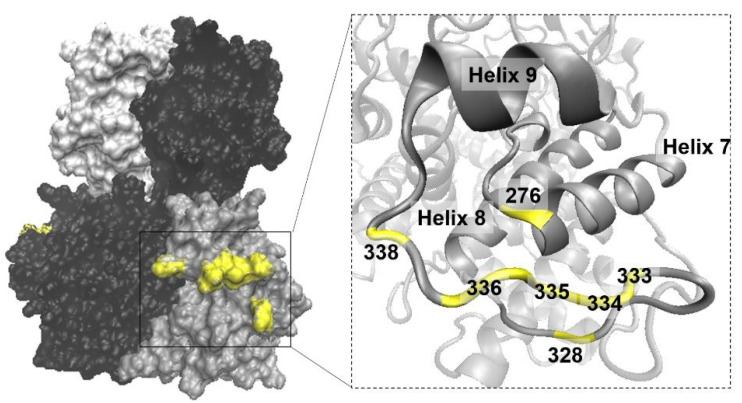
Three-dimensional analysis of the position and secondary structure of the AHSV VP7 “276” region. The AHSV VP7 trimer model is depicted on the **left** with monomers coloured in grey, black, or white. Amino acids targeted for substitution (276, 328, 333–336, and 338) are highlighted in yellow. The “276” region is magnified on the **right**, depicting the secondary structure of the region, and amino acid positions are illustrated and highlighted in yellow.

The abolishment of VP7 self-assembly into crystalline particles evident in the VP7-276 construct provided us with the opportunity to pinpoint the residues responsible for this process. To understand the nature of these trimer–trimer interactions, the “276” region of WT AHSV VP7 was modelled (Figure 5). The modelling of the secondary structure of the “276” region revealed that native residues 328 to 338 in this region form a highly surface-exposed extended loop that connects helix 8 and helix 9 of the VP7 trimer bottom domain, while residue 276 forms part of helix 7 (Figure 5). This loop, as with most of the AHSV VP7 loops, is distinctly different from the corresponding BTV VP7 domain, as reported previously [47].

**Figure 6:**
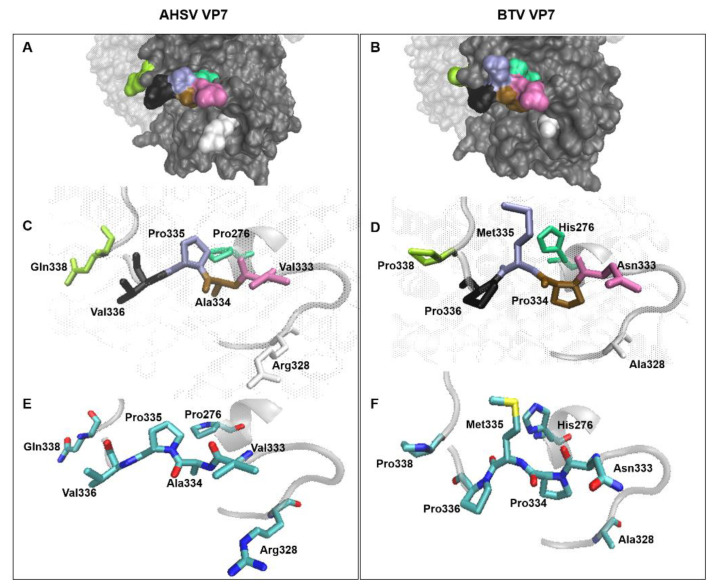
Modelling and three-dimensional comparison of the “276” region of wild-type AHSV and BTV VP7. Targeted amino acids are coloured individually in surface comparisons (**A,B**) and side-chain comparisons of each residue (**C,D**) for AHSV and BTV respectively. Residue properties are compared between AHSV and BTV VP7 via colour elements in each side-chain (blue = nitrogen; red = oxygen; cyan = carbon; yellow = sulphur) (**E,F**).

To further study the nature of this newly identified trimer–trimer interaction region, the physico-chemical properties of the amino acids involved, and their substitutions, were examined. The substitutions made in VP7-276 include Pro_276_His, Arg_328_Ala, Val_333_Asn, Ala_334_Pro, Pro_335_Met, Val_336_Pro, and Gln_338_Pro. It is unknown if all seven of the substitutions made in this construct are responsible for the abolishment of trimer–trimer interactions. Therefore, the whole segment was analysed, and the contribution of each amino acid substitution was considered individually. Figure 6 shows models for the surface and side-chains of the residues of the “276” regions of AHSV and BTV VP7 for comparison, and Table 2 records the chemical and functional changes brought about by these substitutions. From Figure 6 A,B, it is evident that residue 276, in both AHSV and BTV VP7, is largely buried and separate from the remaining residues in this region. The substitution of proline to histidine at this position is generally unfavourable given the unique properties of each amino acid (Table 2). Proline is often found in very tight turns in protein structures, and its function in α-helices is to introduce kinks to allow for a polypeptide chain to change direction, as it cannot adopt a normal helical conformation [53]. Therefore, the proline located in AHSV VP7 at position 276, at the beginning of helix 7, is likely a structural feature. Furthermore the corresponding BTV residue, a histidine, also appears to be involved in forming a kink in the beginning of helix 7 and is unlikely to contribute to exterior functional interactions [53] (Figure 6C–F). Proline and histidine at position 276 in both AHSV and BTV VP7 are, therefore, unlikely to be functioning in the interactions caused by region 276.

Next the residues that make up the exposed loop of VP7-276, i.e., positions 328, 333 to 336, and 338, were closely examined. When comparing the overall changes made to the side-chains in the loop, it was noted that the side-chains of (i) Arg_328_, (ii) Val_336_, and (iii) Gln_338_ of AHSV VP7 protrude outwardly to the aqueous environment, with their side-chains available for external interactions (Figure 6E,F and Table 2). Arginine (Arg_328_) frequently plays an important role in structure and function as, being polar and positively charged, it frequently forms salt bridges with other negatively charged residues (such as glutamate) to create stabilising hydrogen bonds that can form stable interactions. In addition, the long side-chain of arginine often allows for involvement in protein binding sites with neighbouring residues. Valine (Val_336_) is central to the 276 loop region, and highly exposed to the aqueous environment. Despite its non-reactive hydrophobic side-chain, valine can play a role in substrate recognition and hydrophobic interactions. Glutamine (Gln_338_) is a polar amino acid usually found at the surface of a protein. Glutamines are quite frequently involved in protein active or binding sites, and their polar side-chains are good for interactions with other polar or charged atoms. In stark contrast to these AHSV VP7 residues, the side-chains of the corresponding BTV residues (i.e., Ala_328_, Pro_336_, and Pro_338_) are not free for interaction in the exterior aqueous environment. Specifically, both rings of Pro_336_ and Pro_338_ (in the Val_336_Pro and Gln_338_Pro substitutions) are very non-reactive, and together with its difficulty in adopting many protein main-chain conformations, proline is very rarely involved in protein active or binding sites. In addition, the small, non-polar side-chain of BTV Ala_328_ (Arg_328_Ala substitution) is also non-reactive and rarely involved in protein function. 

Looking at the consequence of the amino acid substitutions at these positions, the polar-to-hydrophobic changes, i.e., Gln_338_Pro and Arg_328_Ala, were considered likely to play a large role in the functioning of the trimer–trimer interactions that occur in this region. If this were the case, the forces responsible for AHSV VP7 trimer–trimer interactions would be electrostatic. To further examine the role of these polar amino acids in AHSV VP7 trimer–trimer formation, VP7 mutants containing single amino acid substitutions, i.e., Arg_328_Ala and Gln_338_Pro, as well as a double mutant, i.e., Arg_328_Ala/Gln_338_Pro, were generated via baculovirus expression as described above. However, it was found that all constructs continued to self-assemble into crystalline particles.

A change from valine to asparagine (Val_333_Asn) is typically unfavourable as asparagine is polar and prefers to substitute for other polar amino acids (Figure 6 and Table 2). Asparagine is frequently involved in protein active or binding sites, and its polar side-chain is good for interactions with other polar or charged atoms. Interestingly, the polar asparagine is present in BTV VP7, i.e., the protein that does not possess trimer–trimer interactions. AHSV VP7 has a hydrophobic valine at this position. Given that asparagine is polar and usually prefers to be exposed to the aqueous environment, it may be concluded that the interactions that drive trimer–trimer formation are hydrophobic in nature or that residues at positions 333 do not play a role in trimer–trimer interactions (Figure 6E,F). 

Upon examination of the positioning and properties of AHSV Pro_335_ and Ala_334_ (Figure 6 and Table 2), it can be assumed that these residues do not play a role in exterior protein–protein interactions due to their innate small, non-polar, and non-reactive inward-facing side-chains. These residues are, therefore, unlikely to contribute to any exterior protein interactions, but rather, contribute to the integrity of the loop. The Pro_335_Met and Ala_334_Pro substitutions most likely did not alter the structural integrity of the loop, as the BTV corresponding methionine and proline side-chains are also hydrophobic, non-reactive and positioned inwardly toward the core. In addition, these substitutions do not have any significant functional or chemical feature changes, as indicated in Table 2. That is not to say, however, that the functioning of these residues together is not responsible for the functioning of the 276 region. 

**Figure 7:**
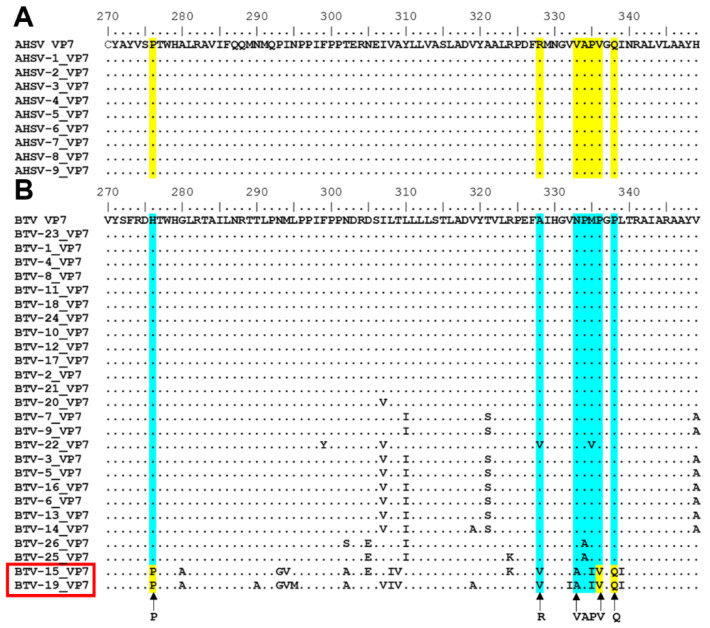
Amino acid sequence conservation analysis of the “276” region among AHSV (**A**) and BTV (**B**) serotypes: Amino acid sequence alignment of AHSV VP7 serotypes 1 to 9 (**A**) and BTV VP7 serotypes 1 to 26 (**B**), with highlighted positions of the “276” region, i.e., positions 276, 328, 333–336, and 338 in yellow for AHSV VP7 and blue for BTV VP7. The consensus sequence of AHSV and BTV VP7 are shown as reference sequences. AHSV VP7 residues are indicated with arrows in (**B**).

The results, thus far, have indicated that AHSV VP7 insolubility can be attributed to some or all seven amino acids of the “276” region located at the C-terminal end of the protein, i.e., Pro_276_, Arg_328_, Val_333_, Ala_334_, Pro_335_, Val_336_, and Gln_338_. The conservation of the “276” region of AHSV and BTV VP7 was analysed next, and compared by performing amino acid sequence alignments. It was found that the “276” region of AHSV VP7 is 100% identical in all nine AHSV serotypes (Figure 7A), indicating that VP7 crystalline particle formation is likely conserved across all AHSV strains. The “276” region of BTV VP7 was highly conserved, except in the most divergent BTV serotypes BTV-15 and BTV-19 (Figure 7B). Interestingly, some residues in this region of BTV-15 and BTV-19 had some homology to the corresponding AHSV VP7 residues, i.e., Pro_276_, Val_336_, and Gln_338_ (Figure 7A).

## 4. Discussion

AHSV VP7 has been shown to self-assemble into unique hexagonal crystalline particles in a process that is independent from host trafficking pathways and the presence of other AHSV proteins [9]. Here, we show that this AHSV VP7 self-assembly process is driven by unique trimer–trimer interactions that are governed by amino acid residues in the C-terminus bottom domain of VP7. To identify the candidate residues that drive the unique AHSV trimer–trimer interactions, surface AHSV VP7 amino acids that differed physiologically from the soluble BTV VP7 amino acids were selected and mutated here. Six mutant AHSV VP7 proteins were generated using the baculovirus expression system, and their ability to form trimer–trimer interactions was tested by analysing protein solubility and crystalline particle formation. The trimer–trimer interactions were successfully targeted and abolished, resulting in the generation of a novel, fully soluble AHSV VP7 protein.

The crystallization of any protein requires the packaging of molecules in a very highly ordered manner, i.e., via the process of oligomerisation. Therefore, the process of AHSV VP7 crystalline particle formation must require precise and uniform packaging of VP7 trimers into a lattice to form a crystalline array. Interestingly, the rotavirus nucleocapsid protein, VP6, which also forms hexameric ring-like trimers on the surface of the inner capsid (much like VP7 of AHSV) self-assembles into hexamers, hexagonal lattices, and tubular and spherical particles [54]. Protein–protein contacts in crystalline particles are complex and involve a precise balance of specific and non-specific flexible interactions. Specific interactions, as targeted here, include hydrogen bonding and electrostatic interactions that involve the participation of flexible amino acid side-chains on the protein surface during crystal lattice formation. Nonspecific interactions may include van der Waals forces and hydrophobic interactions, which may also be influenced by amino acid side-chains. The disruption of any of these forces is, therefore, likely to have a somewhat significant effect on crystal lattice ordering and/or innate protein folding in general. In the case of our mutant proteins that formed disordered aggregates, i.e., VP7-131 and VP7-172, we find that the regions targeted are located along the side of the trimer. The regions targeted in both constructs overlap the interface between monomers. Therefore, it is likely that changes in this area of the trimer would result in the destabilisation of monomer–monomer contacts at this interface, thereby explaining the lack of stable trimers and the formation of disordered aggregates of VP7-131 and VP7-172.

As for the constructs that formed crystalline particles, VP7-136 and VP7-193 are likely resultant from the alternative layering of VP7 to form altered crystalline particles. When examining the position of the regions of VP7-136 and VP7-193, it is noted that both regions are located on the top of the trimer. Specifically, VP7-193 residues are found at the apex of the trimer. Given that the crystalline particles formed by this construct differ from WT VP7, it can be concluded that normal VP7 layering in the crystalline lattice involves some top-to-top/-bottom/side-to-side stacking, as this stacking is disrupted in VP7-193. Given that crystalline particles are still formed by this construct, it can be assumed that the top-to-bottom/top-to-top stacking of trimers is disrupted, thus resulting in thinner, longer spindle-like particles. These observations further indicate that the interactions that drive crystal formation must be located on the side of the trimer as these side-by-side interactions (still intact in VP7-193) are still in play. The region altered in the VP7-136 construct, which is located around the outside of the top of the trimer, was shown to form more disordered crystalline particles. Given that the top of the trimer is involved in the correct stacking of VP7 to form an ordered lattice, the stacking of VP7-136 is, therefore, also affected. The altered distribution from VP7-193, which forms longer, thinner crystalline particles, indicates that the residues targeted in VP7-136 must be interfering with VP7 side-by-side interactions to a small extent.

The results of this study indicated that the trimer–trimer interactions that were suggested to drive AHSV VP7 self-assembly into crystalline particles were persistent, and despite numerous modifications, VP7 managed to self-assemble into particulate structures. Upon analysis of the self-assembly abilities of the VP7 constructs, it could clearly be observed that only one mutant, VP7-276, did not self-assemble into any morphological entities, as evidenced by its ability to form highly soluble trimers that remain evenly distributed throughout the cell—much like BTV VP7 [42]. These results indicated that the proposed trimer–trimer interactions targeted in VP7-276 were successfully disrupted. In turn, these results further confirm that AHSV trimer–trimer interactions are, indeed, responsible for AHSV VP7 self-assembly into crystalline particles. The combination of the AHSV VP7 amino acids Pro_276_, Arg_328_, Val_333_, Ala_334_, Pro_335_, Val_336_, and Gln_338_ are therefore responsible for the unique ability of AHSV VP7 to self-assemble into crystalline particles—likely through hydrophobic interactions. These residues were also shown to be fully conserved across all AHSV serotypes, indicating that crystalline particle formation is likely conserved in all serotypes. Examination of the positioning of these residues on the AHSV VP7 trimer revealed that these residues are located on the side of the trimer, in the bottom domain of VP7, leaving the top domain free for further interaction with VP5 and VP2. This group of residues is located on a region of the trimer that protrudes extensively and is very highly exposed to the aqueous environment. The positioning of the 276 region, therefore, further suggests that VP7 trimers are stacked primarily side-by-side in the crystalline lattice.

As part of our original hypothesis, it was stated that the proposed trimer–trimer interactions that drive AHSV VP7 crystal formation are unrelated to the side-by-side trimer–trimer interactions that are required for CLP formation, as previously described for BTV [53]. For this reason, the ability of VP7-276 to assemble into CLPs was tested. Additionally, since the modifications of VP7-276 resulted in a soluble protein that did not self-assemble into crystalline particles, it was important to test whether these changes interfered with the native orbivirus side-by-side trimer–trimer interactions required for CLP formation for downstream applications. The results showed that VP7-276 could interact with VP3 to form CLPs, and this association was more prevalent than with WT VP7. These results confirm previous reports stating that improved VP7 solubility is the key to efficient CLP formation [34]—likely because there are more freely available VP7 trimers available to interact with VP3, as evidenced in this study.

The results indicate that the majority of WT VP7 is sequestered into crystalline particles, and the CLP formation of WT VP7 with VP3 is undetectable compared to the soluble VP7 version. Importantly, the results showed that the AHSV VP7 trimer–trimer interactions that drive crystalline particle formation are not related to the interactions required for orbivirus CLP formation. This offers a possible explanation as to how this trait has remained conserved for AHSV, in that the interactions are not related to CLP formation and, therefore, the fitness of the virus. A possible explanation for the conservation of AHSV VP7 crystalline particle formation is that a large amount of AHSV VP7 is not required during core assembly, as the availability of other structural proteins such as VP3, VP2, or VP5 may be limited. That being the case, the conservation of VP7 crystalline particle formation would not be due to positive selection from any benefits, but rather, stabilising selection. In contrast, given that AHSV pathogenicity and disease are more severe than BTV, it could be possible that the AHSV VP7 crystalline particles contribute to AHSV cellular damage and pathogenesis. With the availability of a soluble VP7, these hypothesises can now be tested.

The role of virus protein aggregation in human pathology has been studied. The most recent example of which is SARS-CoV-2 and SARS-CoV. Using several computational tools, 10 aggregation-prone proteins in the SARS-CoV-2 reference strain were identified [10]. The aggregation of these predicted aggregation-prone SARS-CoV-2 proteins, including spike protein peptides, NSP6 protein peptide, ORF10, and NSP1, was confirmed in vitro, and it was also revealed that NSP11 aggregates were toxic to mammalian cells [11]. These amyloid aggregates were suggested to possibly contribute to the severe neurological manifestations observed in COVID-19. In addition, it was found that the expression of SARS-CoV-2 ORF8 formed aggregates in lung epithelial cells, suppressed the basal expression of several antiviral molecules, and inhibited IFNγ-induced antiviral gene expression in these cells [12]. A previous study showed that aggregated ORF8b in SARS-CoV induces endoplasmic-reticulum stress, lysosomal damage, and the activation of autophagy [55]. Aggregation has also been reported for SARS-CoV M protein [56] and the C-terminal end of protein E [57]. Other examples of the association of aggregates with human disease include Hepatitis B virus X protein aggregates, which contribute to liver pathogenesis [14] and the PB1-F2 accessory protein of influenza A virus, which assembles into amyloid structures that disrupt the cell membrane and cause cell death in U937 and A549 cells [15]. Therefore, the aggregation of proteins is linked with human disease in a variety of ways. When infected with a virus, it can be expected that the aggregation of viral proteins may result in damage to the host cells. An effort must, therefore, be made to better understand the effects of viruses on the host protein-homeostasis system toward helping future therapeutic efforts.

The modelling of the “276” region revealed that the residues involved form a highly exposed extended loop that is highly variable from BTV VP7 [47]. Loops are mainly defined as being any polypeptide segment exposed to the surface, whose surface residues are usually involved in fewer intramolecular side-chain interactions than their buried counterparts. There are, therefore, lower constraints on loop-sequence conservation, which makes surface loop sites evolve more rapidly and independently than the protein core [58]. For this reason, loop lengths are often more conserved between unrelated proteins than loop structural and/or sequence similarity [59]. The functional differences that exist between members of the same protein families are usually a consequence of differences in structure on the protein surface. Structural variability in a given fold results from substitutions, insertions, and deletions of residues between members of the family [58]. These changes are usually found on exposed loop regions, thus often allowing loops to determine the functional specificity of a given protein contributing to binding sites [60]. It is these factors that likely lead to the changes in residues that cause AHSV and BTV VP7 to deviate in surface properties. Loops have previously been shown to play a role in recognition sites [61], protein–protein interactions, dimerisation, and ligand binding [62,63]. Here, the “276” loop has been shown to play such a role in AHSV VP7 trimer–trimer interaction.

Given that the amino acid differences between AHSV and BTV VP7 that drive crystalline particle formation are simple, the question of why this trait in AHSV is conserved remains unanswered. Now that a version of AHSV VP7 that does not assemble into crystalline particles exists, we will generate a recombinant AHSV virus that does not form crystalline particles via reverse genetics and examine the effect and role of crystalline particle formation on AHSV replication and virulence. 

## 5. Conclusions

In this study, we generated a fully soluble AHSV VP7 protein by targeting residues that differ from the soluble BTV VP7 counterpart. We found that one region on the VP7 trimer surface is responsible for trimer–trimer interactions, which drive the formation of the unique AHSV-specific VP7 crystalline lattice. From these results, we concluded that this soluble AHSV VP7 is capable of forming AHSV CLPs, confirming that the self-assembly of VP7 into insoluble particles likely negatively impacts AHSV CLP yield. This potentially results from the preferential interaction of AHSV trimers with one another, through stronger interactions generated by the “276” region over the native VP7-VP3 interactions required for CLP assembly, thus leaving very few AHSV VP7 trimers available for incorporation into cores.

Much work has gone into the generation of a soluble AHSV VP7 specifically for vaccine research and development [34,46,51,64]. For instance, the insolubility of AHSV VP7 has hindered vaccine strategies such as the addition of AHSV VP7 in subunit vaccines, which has been proven to boost cellular immunity and the overall efficacy of VP2 and VP5 subunit vaccines [42,43]; the insertion of immunogenically important peptides into the top domain of AHSV VP7 trimers has been proven to be a viable vaccine strategy, provided VP7 remains soluble [46]; and the generation of AHSV VLPs as a vaccine strategy has not been possible due to VP7 insolubility [35]. With the availability of a soluble VP7 here, such applications become achievable, and a soluble AHSV VP7 can have a significant impact on AHSV VLP vaccine development.

## 6. Patents

The work reported in this manuscript has resulted in the following patent: Modification of African Horse sickness virus VP7 protein, Republic of South Africa, patent number ZA201706967B.

## Figures and Tables

**Table 1:** Sequences of dsDNA fragments depicting mutations (underlined), insertions (bold), deletions (---), and 15 bp homologous flanking sequences (grey highlight).

Modified VP7	dsDNA Fragment
**VP7-45**	TATAATGGTTTAACATTACGAGGAGTATCGATGAGGCCAACCACCTTAGCAGAACGAAATGAAATGTTTTTTATGTGTACTGATATGGTTTTAGCGGCGCTGAACGTCCAAATTGGGCCGATTTCACCAGATTATACTCAACATTTGGCAACTGTGGGA
**VP7-131**	ACGGGGCCTTATGCAGAAGCGGAGGAGGTGCAACAATCTGGCAGATATTACGTACCGCAAGGTCGAACGCGTGGTGGGGTGATCAATTCAAATATTGCAGAAGTGTGTATGGATGCAGGTGCTGCGGGACAGGTCAATCAGCTGCTACAGGGTCGTAAT---GAT---CCCGTCATGATCTATTTCGTTTGGAGAAGATTGCGTATATTTTGTGATCCTCAAGGTGCGTCACTTGAGAGCGCTCCAGGAACTTTTGTCACCGTTGATGGAGTAAATGTTAGGGCTGGACGCGTCGTCGCATGGAAT
**VP7-136**	GGAGCGGTTGAGGTGTTCCAATCTGGCAGATATTACGTAC**GCG**CAGCTCAAGCAGTAACTGGTGTATACATCAATTCAAATATTGCAGAAGTGTGTATGGATGCAGGTGCTAGAGGACAGGTCAATGCGCTGCTAGCCCCAAGGAGGGGAGACGCAGTCATGATCTATTTCGTTTGGAGAAGATTGCGTATATTTTGTGATCCTCAAGGTGCGTCACTTGAGAGCCAAGCGGGAACTACTGTCACCGTTGATGGAGTAAATGTTGCAGCTGGAGATGTCGTCGCATGGAATACTATTGCACCAGTGAATGTTCATAATCCGACACAACAGAATTCAATTTTACAGTTT
**VP7-172**	GCGGGACAGGTCAATCAGATATTTCAG*GGT*CGTAAT---GAT---CCCGTCATGATCTATTTC
**VP7-193**	GGAGACCGTTGCGTAACTTTTGTATGGCGCAAGGTAATTCACAGGAGAGCGCTCCAGGA
**VP7-276**	ATGCGTATGTCTCTCACACTTGGCACGCATTACGCGCTGTCATTTTTCAGCAGATGAATATGCAGCCTATTAATCCGCCGATTTTTCCACCGACTGAAAGGAATGAAATTGTTGCGTATCTATTAGTAGCTTCTTTAGCTGATGTGTATGCGGCTTTGAGACCAGATTTCGCGATGAATGGTGTTAATCCGATGCCAGGGCCGATTAACAGAGCTCT

**Table 2:** Substitutions made in the 276 region and their resulting changes in chemical and functional features. Asterix (*) indicates significant feature changes.

Substitution	Amino Acid Property	Charge	Side-Chain	Hydrophobicity ^1^	Size	Surface Preference	Hydrogen Bonding?
Pro_276_His	Non-polar → Basic	0 → +	Cyclic → Aromatic	Phob → Phil	Small → Large	Surface → Surface	No → Yes
* Arg_328_Ala	* Basic → Non-polar	* + → 0	Charged → Aliphatic	Phil → Phob	* Large → Small	Surface → Buried	* Yes → No
Val_333_Asn	* Non-polar → Polar	0 → 0	Acyclic → Acyclic	Phob → Phil	Medium → Medium	Buried → Surface	* No → Yes
Ala_334_Pro	Non-polar → Non-polar	0 → 0	Cyclic → Acyclic	Phob → Phob	Small → Small	Buried → Surface	No → No
Pro_335_Met	Non-polar → Non-polar	0 → 0	Cyclic → Acyclic	Phob → Phob	Small → Large	Surface → Buried	No → No
Val_336_Pro	Non-polar → Non-polar	0 → 0	Acyclic → Cyclic	Phob → Phob	Medium → Small	Buried → Surface	No → No
* Gln_338_Pro	* Polar → Non-polar	0 → 0	* Acyclic → Cyclic	Phil → Phob	* Large → Small	Surface → Surface	* Yes → No

^1^ Changes to/from hydrophobic (Phob) and/or hydrophilic (Phil).

## Data Availability

The data generated and used in this study are available upon request from the corresponding author.

## Supplementary Materials

The following supporting information can be downloaded at: https://www.mdpi.com/article/10.3390/v14081624/s1, Figure S1: Solubility assay of modified VP7 proteins; Figure S2: Intracellular distribution of VP7 mutant proteins via indirect immunofluorescence microscopy; Video S1: (A) and (B): Colocalisation analysis of WT VP7 with VP3.
