# Supplementary figures and images for: Generation of a Soluble African Horse Sickness Virus VP7 Protein Capable of Forming Core-like Particles
Source: Viruses. 2022 Jul 26;14(8):1624. doi: 10.3390/v14081624 (PMC9331310; doi:10.3390/v14081624)

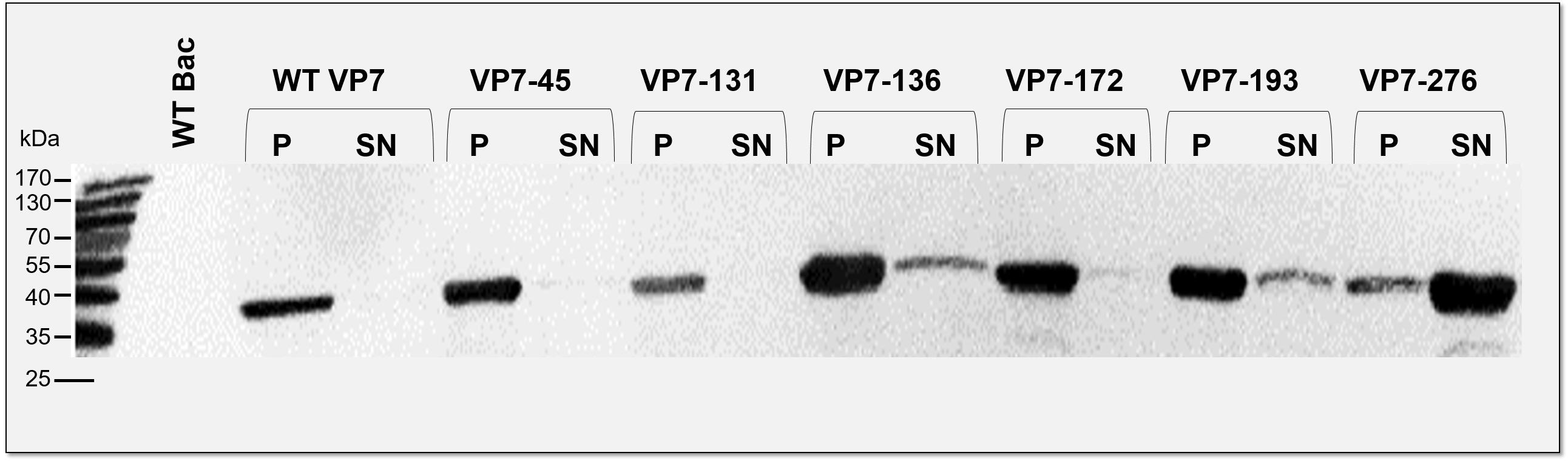

Supplement: Supplementary file 1 [file viruses-14-01624-s001.zip › Figure S1.tif]

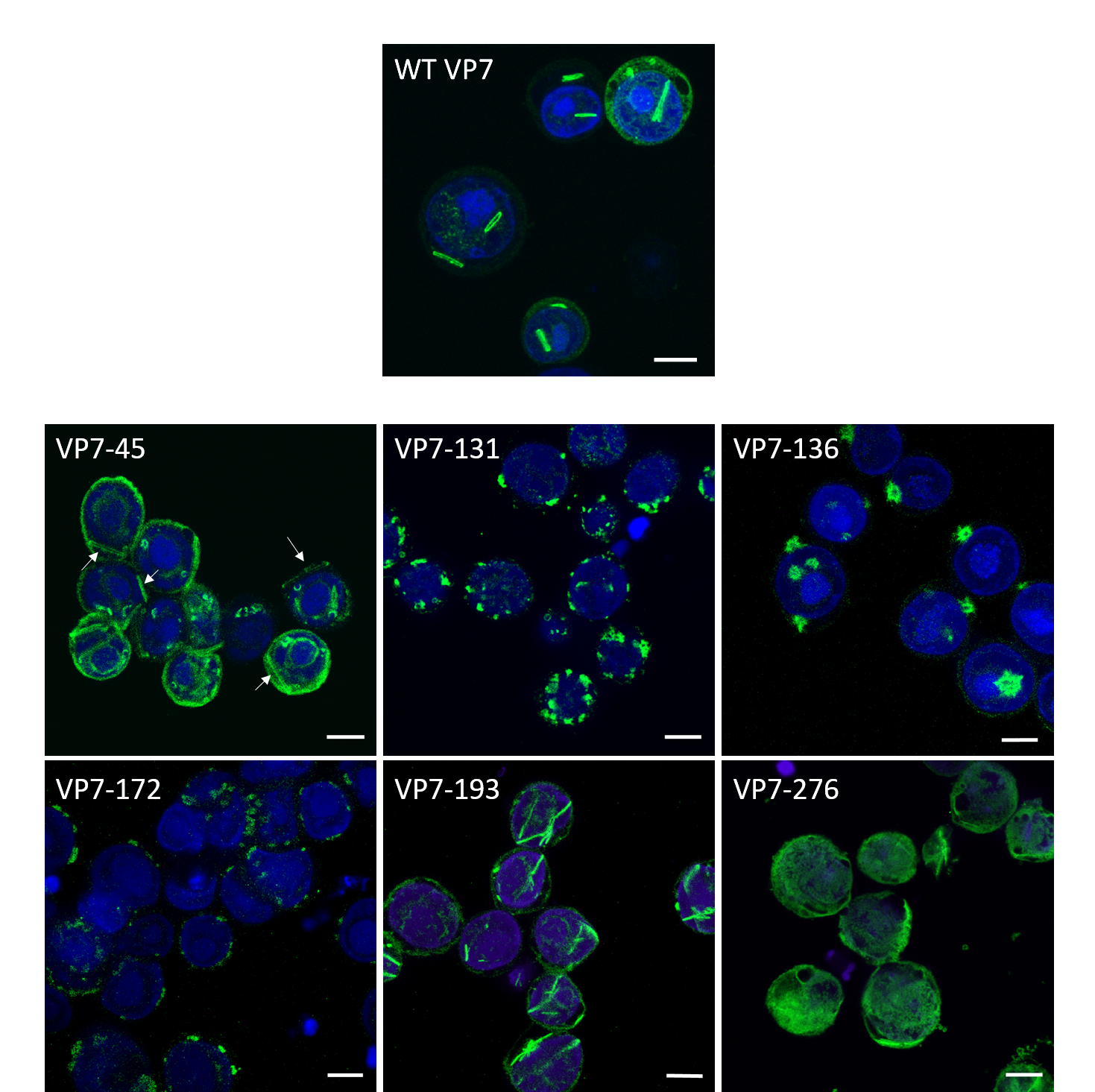

Supplement: Supplementary file 1 [file viruses-14-01624-s001.zip › Figure S2.tif]
